# Pre-exposure Prophylaxis With Various Doses of Hydroxychloroquine Among Healthcare Personnel With High-Risk Exposure to COVID-19: A Randomized Controlled Trial

**DOI:** 10.7759/cureus.20572

**Published:** 2021-12-21

**Authors:** Fibhaa Syed, Muhammad Hassan, Mohammad Ali Arif, Sadia Batool, Rauf Niazi, Ume e Laila, Sadia Ashraf, Junaid Arshad

**Affiliations:** 1 Internal Medicine, Shaheed Zulfiqar Ali Bhutto Medical University, Islamabad, PAK; 2 Neurology, Shaheed Zulfiqar Ali Bhutto Medical University, Islamabad, PAK; 3 Cardiology, Shaheed Zulfiqar Ali Bhutto Medical University, Islamabad, PAK

**Keywords:** hydroxychloroquine., healthcare personnel, covid-19, pre-exposure prophylaxis, sars-cov-2

## Abstract

Objective

This trial aimed to evaluate the safety and efficacy of pre-exposure prophylaxis (PrEP) with various hydroxychloroquine (HCQ) doses against a placebo among healthcare personnel (HCP) with high-risk exposure to coronavirus disease 2019 (COVID 19).

Methods

A phase II, randomized, placebo-controlled trial was conducted including 200 subjects with no active or past severe acute respiratory syndrome coronavirus 2 (SARS-CoV-2) infection (antibody testing and reverse transcription-polymerase chain reaction (RT-PCR) were taken at the time of enrollment). Subjects of experimental groups one to three received HCQ in various doses and the control group received a placebo. The study outcomes in terms of safety and efficacy were monitored. Participants exhibiting COVID-19 symptoms were tested for SARS-CoV-2 during the study and by the end of week 12 with RT-PCR or serology testing (COVID-19 IgM/IgG antibody testing).

Results

Out of the total participants, 146 reported exposure to a confirmed COVID-19 case in the first month, and 192 were exposed by week 12 of the study. Moreover, the precautionary use of personal protective equipment (PPE) significantly varied; initially more than 80% of the exposed HCPs were not ensuring PPE being used by the patients treated by them, which gradually developed over time. Mild treatment-related side effects were observed among the interventional and placebo arm patients. There was no significant clinical benefit of PrEP with HCQ as compared to placebo (p>0.05).

Conclusion

It is concluded that the PrEP HCQ does not significantly prevent COVID-19 among high-risk HCPs.

## Introduction

Severe acute respiratory syndrome coronavirus 2 (SARS-CoV-2) has by far affected almost all countries. Globally, as of September 7, 2021, there have been about 221,134,742 confirmed cases of coronavirus disease 2019 (COVID-19) and 4,574,089 deaths, as per World Health Organization (WHO) emergency statistics [1]. For the proper management of this pandemic, the most crucial step is the conservation of workforce safety. This outbreak has taken a heavy toll on the frontline healthcare personnel (HCP) as they are three times more likely to be infected than unexposed [2]. Moreover, this pandemic has affected us physically and psychologically; the exposed frontline workers frequently develop depressive symptoms, anxiety, fear, and stress [3-5].

Given the HCP exposed to COVID-19 patients are at high risk of viral transmission [6], the fight against COVID-19 is in continuance and its success relies on the adherence to the preventive measures that are being explored for disease containment. In addition to the quarantine of exposed individuals, prevention strategies also include the use of personal protective equipment (PPE), hand hygiene, case identification, and isolation [7-9]. Numerous drugs are undergoing clinical trials for effective mitigation of SARS-CoV-2 transmission [6,10]. Until now, remdesivir is the only drug approved by the Food and Drug Administration (FDA) to treat COVID-19 [8]. Additionally, dexamethasone also improved the disease outcomes among severe COVID-19 patients [11,12]. 

Furthermore, HCQ, a chloroquine derivate is also identified as a possible prophylactic inhibitor for the entry and post-entry stages of SARS-CoV-2, with better in vitro antiviral activity and safety profile, on the ground of anti-inflammatory as well as antiviral effects [13-15]. The available clinical evidence for the use of HCQ among COVID-19 patients seems insufficient and does not fully prove the effectiveness of this therapeutic modality among severely ill COVID-19 patients [16]. Nevertheless, the promising treatment outcomes observed among mildly infected patients and no environmental implications associated with the drug suggest its therapeutic potential.

Pre-exposure prophylaxis (PrEP) is suggested to be a promising strategy for several infectious diseases [17], but none of the pre-exposure pharmacological drugs have been established for COVID-19 yet. However, this study aims to evaluate the therapeutic safety and efficacy of PrEP with various doses of HCQ among the HCP who are at high risk for COVID-19 [18].

This article was previously posted to the medRxiv preprint server on May 17, 2021 [19].

## Materials and methods

Study design and participants

A Phase II, randomized, placebo-controlled clinical trial (Trial registration: Clinicaltrials.gov, NCT04359537; registered May 1, 2020 - prospectively registered)[20] was conducted at Shaheed Zulfiqar Ali Bhutto Medical University (SZABMU)/Pakistan Institute of Medical Sciences (PIMS). Enrollment began on May 1, 2020, and the intervention continued for a total of 12 weeks. The study protocol was approved by the SZABMU ethical review board (Ref# 1-1/2015/ERB/SZABMU/549; dated April 20, 2020), and written informed consents were acquired from the participants before inclusion. All data generated or analyzed during this study are included in this published article (and its supplementary information files).

All HCP at high risk for COVID-19 exposure, primarily first responders, those performing aerosol-generating procedures, and those working in emergency departments, ICUs, and the departments of general medicine, pulmonology, infectious disease, and isolation wards were included in the study. Active COVID-19 cases, those with existing symptoms like fever, cough, shortness of breath, having prior retinal eye disease, chronic kidney disease (CKD) stage 4 or 5 or undergoing dialysis, glucose-6 phosphate dehydrogenase (G-6-PD) deficiency, recent myocardial infarction (MI) and epileptic subjects were excluded. Additionally, also kept under exclusion were pregnant females, subjects weighing < 40 kg, those having contraindication or allergy to chloroquine/HCQ, those already under the administration of HCQ or cardiac medicines like flecainide, amiodarone, and digoxin, etc., medications with known significant drug-drug interactions like artemether, and lumefantrine, etc., and those causing QT interval prolongation like macrolides, and antipsychotics, etc.

**Figure 1:**
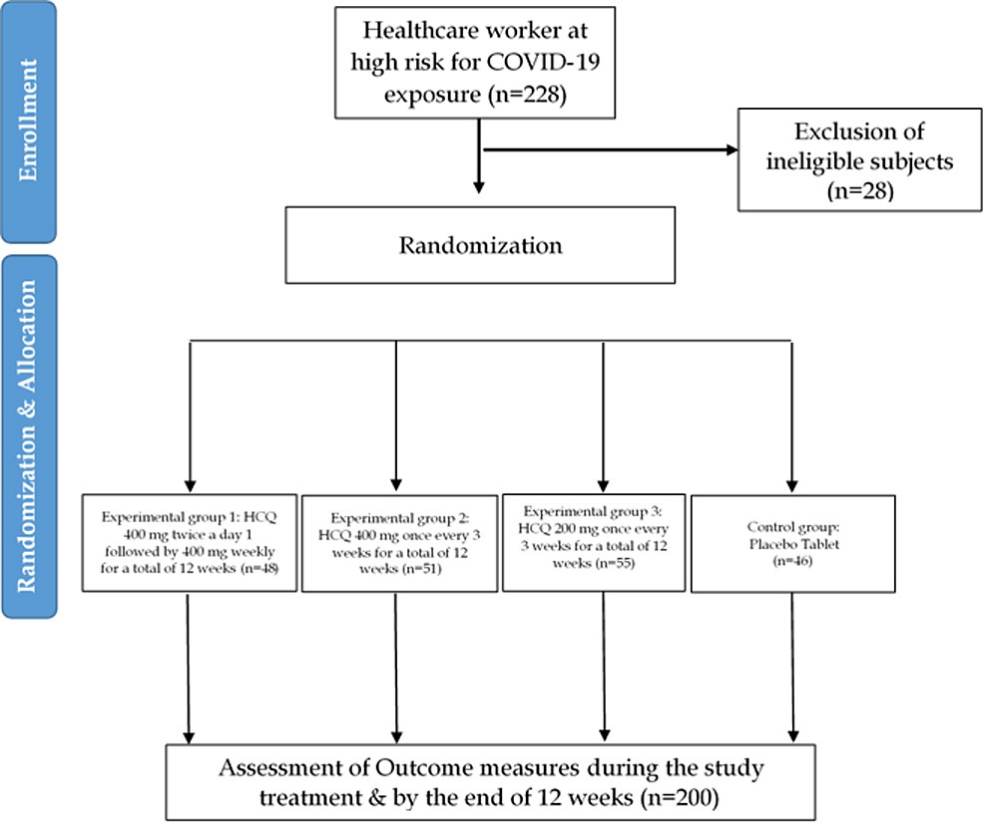
Flowchart of the study

A total of 228 participants were initially enrolled; of them, 28 were ineligible and excluded. Participants fulfilling the eligibility criteria were randomized into the four treatment groups (Figure 1). Group 1 participants (n=48) were intervened with HCQ 400 mg twice a day on day 1 followed by 400 mg weekly. Group 2 (n=51) with HCQ 400 mg once every three weeks, Group 3 (n=55) with HCQ 200 mg once every three weeks, and the Control Group received a placebo (n=46).

Assessments and follow-up procedure

The baseline characteristics of all participants, including age, gender, role, comorbidities, and drug records, were obtained. COVID-19 related symptoms and adverse events (AEs) from the drug were self-reported by the enrolled participant during the study period. The COVID-19 exposure and preventive practices were monitored on a monthly basis. Disease severity was assessed through an ordinal scale (score 1-5), i.e. no illness, illness with outpatient observation, hospitalization (or post-hospital discharge), hospitalization with ICU stay (score=4), and death from COVID-19. All participants exhibiting COVID-19 symptoms were tested for SARS-CoV-2 during the study and also by the end of week 12, with RT-PCR or IgM and IgG serology (as per accessibility).

Outcomes

The primary endpoint was to evaluate the COVID-19-free survival among the participants by the end of the study. The secondary endpoints were to evaluate the proportion of RT-PCR positive COVID-19 cases, the role of exposure and preventive practices, frequency of COVID-related symptoms, treatment-related side effects, the incidence of all-cause study medicine discontinuation, and maximum disease severity during the study treatment.

Statistical methods

The continuous variables were summarized as means and standard deviations (SD) and categorical variables as frequencies and percentages. A comparative analysis was performed between the experimental groups. Chi-square test and one-way ANOVA were used to compare the demographic and clinical characteristics across treatment groups. A p-value <0.05 was considered significant.

## Results

The baseline characteristics of the enrolled HCP are shown in Table 1. The trial included 109 (54.5%) male participants, and the mean age was 30.63 ± 8.07 years. The majority of them were doctors and had no comorbid conditions.

**Table 1:** Demographic characteristics of the studied population (n=200) *Values are given as n(%) or Mean ± SD

Variable		Experimental			Control Group (n=46)	p-value
		Group 1 (n=48)	Group 2 (n=51)	Group 3 (n=55)		
Age (years); Mean ± SD		30.40±7.764	28.20±4.486	32.02±9.517	31.91±9.13	0.057
Gender	Male	24(50)	25(49.0)	37(67.3)	23(50)	0.172
	Female	24(50)	26(51.0)	18(32.7)	23(50)	
Profession	Doctor	32(66.7)	43(84.3)	43(78.2)	25(54.3)	0.050
	Nurse	9(18.8)	4(7.8)	7(12.7)	9(19.6)	
	Technician	1(2.1)	-	2(3.6)	5(10.9)	
	Emergency Staff	1(2.1)	-	1(1.8)	-	
	Emergency Responder	-	2(3.9)	-	-	
	Sanitary Worker	2(4.2)	1(2.0)	-	2(4.3)	
	Security Guard	3(6.3)	1(2.0)	2(3.6)	5(10.9)	
Comorbidities	None	45(93.8)	50(98.0)	48(87.2)	41(89.1)	0.222
	Diabetes	2(4.2)	-	2(3.63)	3(6.5)	
	Hypertension	1(2.1)	1(2.0)	5(9.09)	2(4.3)	
Smoking Status	Smoker	5(10.4)	7(13.7)	7(12.7)	7(15.2)	0.917
	Non-Smoker	43(89.6)	44(86.3)	48(87.3)	39(84.8)	

There was no significant difference in the exposure records between the treatment groups as shown in Table 2.

**Table 2:** Monthly evaluation of COVID exposure and prevention *Values are given as n(%) PPE: personal protective equipment; HCP: healthcare personnel; COVID 19: coronavirus disease 2019

Variables			Experimental			Control Group	p-value
			Group 1	Group 2	Group 3		
Exposed to confirmed COVID-19 case	First Month		35(72.9)	41(80.4)	41(74.5)	29(63.0)	0.285
	Second Month		43(89.6)	49(96.1)	52(94.5)	45(97.8)	0.326
	Third Month		45(93.8)	50(98.0)	51(92.7)	46(100)	0.2
Times Exposed	First Month	1-5	32(91.4)	24(58.5)	32(78.0)	20(71.4)	0.049
		6-10	3(8.6)	15(36.6)	8(19.5)	8(28.6)	
		11-15	-	2(4.9)	1(2.4)	-	
	Second Month	1-5	29(65.9)	21(42.9)	32(61.5)	28(65.1)	0.192
		6-10	14(31.8)	18(36.7)	14(26.9)	13(30.2)	
		11-15	1(2.3)	5(10.2)	4(7.7)	-	
		16-20	-	4(8.2)	1(1.9)	2(4.7)	
		> 20	-	1(2.0)	1(1.9)	-	
	Third Month	1-5	24(53.3)	11(22.4)	22(44.0)	31(68.9)	0.001
		6-10	16(35.6)	16(32.7)	15(30.0)	9(20.0)	
		11-15	2(4.4)	6(12.2)	7(14.0)	2(4.4)	
		16-20	3(6.7)	5(10.2)	3(6.0)	1(2.2)	
		> 20	-	11(22.4)	3(6.0)	2(4.4)	
PPE used by HCP	First Month	Gown	-	-	1(1.8)	1(2.2)	0.01
		Surgical Mask	6(12.5)	1((2.0)	4(7.3)	11(23.9)	
		N95 Mask	1(2.1)	1(2.0)	-	1(2.2)	
		Tyvek^®^ Suit	6(12.5)	10(19.6)	2(3.6)	3(6.5)	
		Gloves + Gown + Surgical Mask	16(33.3)	17(33.3)	17(30.9)	8(17.4)	
		Gloves + Gown + N95 Mask	2(4.2)	11(21.6)	13(23.6)	11(23.9)	
		Gloves + Surgical Mask	7(14.6)	2(3.9)	9(16.4)	2(4.3)	
		None	10(20.8)	9(17.6)	9(16.4)	9(19.6)	
	Second Month	Gloves	-	-	2(3.6)	-	0.002
		Gown	-	-	-	2(4.3)	
		Surgical Mask	1(2.1)	2(3.9)	7(12.7)	5(10.9)	
		N95 Mask	-	1(2.0)	-	1(2.2)	
		Tyvek^®^ Suit	4(8.3)	15(29.4)	5(9.1)	3(6.5)	
		Gloves + Gown + Surgical Mask	18(37.5)	13(25.5)	19(34.5)	11(23.9)	
		Gloves + Gown + N95 Mask	12(25.0)	11(21.6)	14(25.5)	9(19.6)	
		Gloves + Surgical Mask	8(16.7)	6(11.8)	6(10.9)	15(32.6)	
		None	5(10.4)	3(5.9)	2(3.6)	-	
	Third Month	Gloves	-	-	1(1.8)	-	0.151
		Surgical Mask	6(12.5)	3(5.9)	7(12.7)	4(8.7)	
		N95 Mask	2(4.2)	-	-	4(8.7)	
		Tyvek^®^ Suit	12(25.0)	21(41.2)	9(16.4)	12(26.1)	
		Gloves + Gown + Surgical Mask	10(20.8)	13(25.5)	13(23.6)	13(28.3)	
		Gloves + Gown + N95 Mask	14(29.2)	8(15.7)	17(30.9)	11(23.9)	
		Gloves + Surgical Mask	1(2.1)	4(7.8)	4(7.3)	2(4.3)	
		None	3(6.3)	2(3.9)	4(7.3)	-	
PPE used by the patient	First Month	None	42(87.5)	49(96.1)	46(85.2)	42(91.3)	0.008
		Gloves	2(4.2)	-	-	4(8.7)	
		Surgical Mask	4(8.3)	2(3.9)	8(14.8)	-	
	Second Month	None	42(87.5)	46(92.0)	48(87.3)	35(76.1)	0.355
		Gloves	3(6.3)	1(2.0)	2(3.6)	6(13.0)	
		Gown	-	-	1(1.8)	-	
		Surgical Mask	3(6.3)	3(6.0)	4(7.3)	5(10.9)	
	Third Month	None	32(68.1)	40(78.4)	31(56.4)	30(65.2)	0.432
		Gloves	4(8.5)	4(7.8)	4(7.3)	4(8.7)	
		Gown	-	1(2.0)	1(1.8)	-	
		Surgical Mask	11(23.4)	6(11.8)	17(30.9)	10(21.7)	
		Gloves + Gown + Surgical Mask	-	-	2(3.6)	2(4.3)	

Of the total, COVID-19 related symptoms appeared in 54.9% of participants of experimental Group 2, 33.3% of Group 1, 30.4% from the placebo group, and 23.6% from Group 3 (Table 3).

**Table 3:** COVID related symptoms *Values are given as n(%)

Variables		Experimental			Control Group	p-value
		Group 1	Group 2	Group 3		
Symptomatology	Yes	16(33.3)	28(54.9)	13(23.6)	14(30.4)	0.006
	No	32(66.7)	23(45.1)	42(76.4)	32(69.6)	
Fever	Mild	15(31.3)	17(33.3)	9(16.4)	8(17.4)	0.037
	Moderate	2(4.2)	9(17.6)	4(7.3)	5(10.9)	
Cough	Mild	11(22.9)	18(35.3)	6(10.9)	12(26.1)	0.057
	Moderate	5(10.4)	2(3.9)	3(5.5)	1(2.2)	
Shortness of breath	Mild	5(10.4)	1(2.0)	1(1.8)	2(4.3)	0.271
	Moderate	-	1(2.0)	-	-	
	Severe	-	-	1(1.8)	-	
Rhinorrhea	Mild	13(27.1)	15(29.4)	4(7.3)	8(17.4)	0.002
	Moderate	-	3(5.9)	-	-	
	Severe	2(4.2)	-	-	-	
Diarrhea	Mild	3(6.3)	-	2(3.6)	3(6.5)	0.316

No serious AEs were observed. Five participants from experimental Group 1, one from Group 2, three from Group 3, and one from the control group reported treatment-related side effects. The samples were tested for COVID-19, both RT-PCR and serological tests were performed, and the results are shown in Table 4.

**Table 4:** Relationship of management and laboratory test profile of trial subjects *Values are given as n(%) COVID-19: coronavirus disease 2019; PCR: polymerase chain reaction

Variables		Experimental			Control Group	p-value
		Group 1	Group 2	Group 3		
COVID-19 test results (during the study treatment)	Test not done	10(20.8)	12(23.5)	13(23.6)	12(26.1)	0.072
	Positive	15(31.3)	19(37.3)	8(14.5)	7(15.2)	
	Negative	23(47.9)	20(39.2)	34(61.8)	27(58.7)	
Maximum Disease Severity	No Illness	38(79.2)	37(72.5)	49(89.1)	40(87.0)	0.112
	Illness with outpatient observation	10(20.8)	14(27.5)	6(10.9)	6(13.0)	
Treatment-related side effects	Yes	5(10.4)	1(2.0)	3(5.5)	1(2.2)	0.191
	No	43(89.6)	50(98.0)	52(94.5)	45(97.8)	
Drug Discontinuation	Side Effects	2(4.2)	1(2.0)	2(3.6)	1(2.2)	0.897
	Other Reasons	5(10.4)	4(7.8)	3(5.5)	1(2.2)	0.411
PCR Results (end of 12 week)	Positive	3(6.3)	3(5.9)	1(1.8)	3(6.5)	0.492
	Negative	45(93.8)	45(88.2)	53(96.4)	42(91.3)	
	Test not done	-	3(5.9)	1(1.8)	1(2.2)	
Serology Results (end of 12 week)	IgM -ve IgG +ve	10(20.8)	15(29.4)	5(9.1)	8(17.4)	0.183
	IgM +ve IgG +ve	4(8.3)	4(7.8)	4(7.3)	2(4.3)	
	IgM +ve IgG –ve	-	2(3.9)	-	1(2.2)	
	IgM -ve IgG –ve	34(70.8)	30(58.8)	46(83.6)	35(76.1)	

## Discussion

As the HCP are at the highest risk of COVID-19 infection, it is essential to ensure adequate allocation of PPE to alleviate the structural inequities associated with COVID-19. In this study, there is no significant difference in exposure to COVID-19 within the various experimental groups. The rate of exposure to a confirmed COVID case increased by the end of week 12 among the participants of all treatment groups. Although the rate of involvement in the preventive practices wasn't very promising initially, with progressing knowledge, the level of preparedness excelled among the HCP of all study groups. These findings were consistent with a similar randomized trial related to PrEP with the dosage of HCQ in SARS-CoV-2 patients [18]. Moreover, other similar observational studies also indicated an increased risk with the inadequate use of PPE among HCP. Despite adequate PPE use, infection prevention, and control measures, HCP remain at high risk for contracting COVID-19 infection [21].

During the first month of the study, the overall accumulated incidence of COVID-19 among the study participants was 32.02%. The rate of COVID-19 positivity was similar in the HCQ and placebo arms (p=0.072). A subsequent decrease in the COVID-19 incidence was observed by the PrEP with HCQ by the end of week 12, as per the PCR results. Furthermore, most of our study participants (83.6%) tested negative for IgG and IgM antibodies by the end of 12 weeks. Moreover, the infection rate was lowest among the participants treated with the low drug dose. Grau-Pujol et al., in their study, concluded that PrEP with low doses of HCQ is safe and effective [22]. A randomized clinical trial by Abella et al. reported a similar COVID-19 incidence rate among the participants of HCQ and placebo arms (p > 0.99) [23]. Likewise, Boulware et al. suggested no significant difference in the incidence of COVID-19 after the treatment with HCQ than those given placebo only, with additional gastrointestinal and neurologic side effects among the participants of the interventional arm [18].

In this trial, the types and frequency of symptoms reported were similar to those in previous studies involving HCP [24,25]. It was also observed that the treatment-related side-effects were comparatively more evident among the participants of the experimental arms than placebo. However, none of the participants required hospitalization, ICU care, or died from COVID-19 in any study groups. A majority reported no illness in response to the provided treatment, and a few had a mild illness. Our safety data is similar to a randomized clinical trial by Lim et al. indicating a higher rate of AE observed among the intervention arm patients than those receiving placebo [25]. Moreover, five of the present study participants discontinued the HCQ prophylaxis due to side effects and one in the placebo arm, which is consistent with a similar study by Grau-Pujol et al. [26]. 

Adding to the existing literature, this trial provided a detailed analysis of the monthly exposure history, intensity of exposures, and preventive practices of the enrolled HCP. However, we acknowledge the limitations. The small sample size for assessing the efficacy of PrEP with HCQ at the initial stages of analysis among the HCP was one of the major limitations. Based on the findings, the incidence rate of SAR-CoV-2 in HCP declined from the initiation of the study till the end of the analysis. Further large-scale prophylaxis trials are required to investigate the antiviral activity of varying HCQ dosing and the differential impact of each therapeutic agent on the body’s biochemical profile and the overall disease incidence.

## Conclusions

In conclusion, there was no significant reduction in the SARS-CoV-2 transmission with PrEP administration of HCQ among the enrolled HCP. As far as the disease severity is concerned, none of the participants were severe/critical enough to require hospitalization and ICU care, and none of them died.
